# Relation between Halogen Bond Strength and IR and NMR Spectroscopic Markers

**DOI:** 10.3390/molecules28227520

**Published:** 2023-11-10

**Authors:** Akhtam Amonov, Steve Scheiner

**Affiliations:** 1 Department of Optics and Spectroscopy, Engineering Physics Institute, Samarkand State University, University blv. 15, Samarkand 140104, Uzbekistan; akhtamul@gmail.com; 2 Department of Chemistry and Biochemistry, Utah State University, Logan, UT 84322-0300, USA

**Keywords:** chemical shift, coupling constant, blue shift, AIM

## Abstract

The relationship between the strength of a halogen bond (XB) and various IR and NMR spectroscopic quantities is assessed through DFT calculations. Three different Lewis acids place a Br or I atom on a phenyl ring; each is paired with a collection of N and O bases of varying electron donor power. The weakest of the XBs display a C–X bond contraction coupled with a blue shift in the associated frequency, whereas the reverse trends occur for the stronger bonds. The best correlations with the XB interaction energy are observed with the NMR shielding of the C atom directly bonded to X and the coupling constants involving the C–X bond and the C–H/F bond that lies ortho to the X substituent, but these correlations are not accurate enough for the quantitative assessment of energy. These correlations tend to improve as the Lewis acid becomes more potent, which makes for a wider range of XB strengths.

## 1. Introduction

Of all the noncovalent interactions that have been probed over the years, it is the H-bond (HB) that has fostered the largest body of research, stretching back more than a century [1,2,3,4,5,6,7,8,9]. One of the offshoots of this extensive body of work has been the recently developing interest in a set of noncovalent bonds where the bridging proton of the HB is replaced by any of a broad spectrum of other atoms, most commonly from the right side of the periodic table [10,11,12,13,14,15,16]. Some of these analogous interactions are known as chalcogen, pnicogen, and tetrel bonds, depending of course on the particular family from which the bridging atom is drawn [17,18,19,20,21,22,23,24,25,26]. 

Within this grouping of interactions, it is the halogen bond (XB) that has captured the lion’s share of attention. The analogue of the H-bonding A–H··B configuration is altered to A–X··B, where X represents any of the halogen atoms, usually Cl, Br, or I. Although the electronegativity of X works against an overall partial positive atomic charge as is present on H, the molecular electrostatic potential (MEP) surrounding the X is characterized by a positive region directed along the extension of the A–H bond, complemented by a negative equatorial ring. The former is commonly referred to as a σ-hole, which is capable of electrostatically attracting a nucleophile in much the same way as does the bridging proton of an HB [25,27,28,29,30,31,32,33,34]. This coulombic component is supplemented by a stabilizing charge transfer from the nucleophile to the σ*(AX) antibonding orbital, in full analogy to the transfer to the σ*(AH) of the HB, to which is attributed the well-known red shift in the A–H vibrational stretching frequency. 

Again, with reference to the HB, there are a set of spectroscopic indicators of the presence of such a bond, which can be used in some cases to assess the strength of the bond. The aforementioned red shift in ν(AH) is a prime example, where larger shifts are taken as evidence of more powerful HBs [35,36,37,38]. Along with its displacement to lower frequencies, this IR band is typically intensified, the degree of which serves as another indicator of a stronger bond. NMR spectroscopy provides alternative measures of HB strength, particularly through the downfield shift in the signal of the bridging proton.

Because of the high degree of similarity between the HB and XB, one would expect that spectroscopic features ought to serve as useful measures of the strength of the latter bond as well. However, examination of this question has been fragmentary, with little in the way of general trends emerging from past work. A large fraction of the past work concentrated on the effects of the XB upon the electron donor unit [39,40,41,42], meaning it ignored issues arising within the acid. A few cases have been identified where XB formation leads to a red shift in the internal stretching frequency within the halogen donor, usually in small molecules such as a dihalogen [43,44], FX [45,46], CH_3_X [47], or CF_3_X [48], and there are cases where a blue shift has been observed [49], but little systematic work has addressed this issue in larger systems. 

There have been solid state NMR measurements [50] that delved into the effects of the angular characteristics of XB formation upon the NMR spectrum. A number of works have considered very small Lewis acids such as dihalogens [45,46,51,52,53]. NMR coupling constants have been computed for the specific pair of atoms involved directly in the bond [39,54,55,56] but little attention has been paid to the more peripheral nuclei or to the coupling constants within the Lewis acid unit. In connection with larger systems, a certain amount of attention has been drawn toward halobenzenes [57,58,59,60,61,62] where the X atom is connected to a simple phenyl ring. As electron-withdrawing substituents on the ring amplify the X σ-hole, haloperfluorobenzenes have also been the subject of scrutiny [61,63,64,65,66,67]. 

One concept emanating from this work [68,69,70,71] has been that the shielding of the C atom directly attached to I tends to diminish as the XB strengthens and the internal C–I bond grows longer. Indeed, it has been suggested [72] that shielding of this C nucleus might serve as a sort of reporter or measure of XB strength. Some recent calculations echo this idea [73,74] for certain other halogenated systems. A recent paper [75] raised another intriguing proposal that the internal coupling constants within Lewis acids have potential as a monitor of XB strength, at least in the context of fluorinated iodobenzene, where the measurements were made.

The goal of the present work is a systematic evaluation of the ability of various spectroscopic markers to predict and correlate with XB strengths of halobenzene derivatives. To this end, bromobenzene is considered first. The replacement of Br by I is expected to strengthen the XB, which will be further amplified by the perfluorination of the phenyl ring, thus providing a wide range of halogen donor strengths. A variety of bases are considered of both N and O types. The N bases encompass all sp, sp^2^, and sp^3^ hybridizations. H_2_O and OMe_2_ are taken as O bases, followed by the carbonyl O of OCH_2_, OCHNH_2_, and N-methylacetamide, so as to monitor the effects of certain functional groups.

## 2. Results

**Figure 1:**
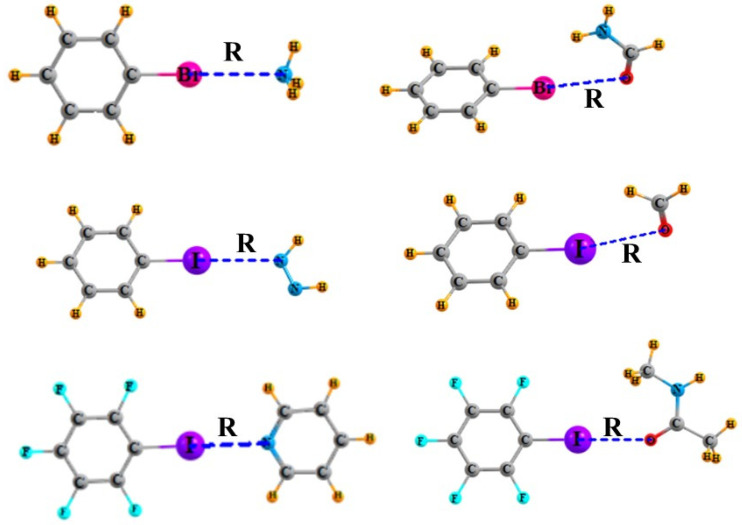
Optimized geometries of several representative complexes. R refers to halogen bond length R(N/O···X).

The optimized geometries of several representative halogen-bonded complexes are displayed in Figure 1, where R refers to the distance from X, either Br or I, to the electron-donating N or O atom of the base. The principal properties of the various complexes are assembled in Table 1 and are organized as follows. The first section refers to Lewis acid bromobenzene PhBr, followed by iodobenzene PhI, and then perfluorinated iodobenzene PhF_5_I in the lowermost section. The internal C–X bond length of each Lewis acid monomer is reported, as well as its vibrational stretching frequency. Within each section, a series of bases are listed, first N-bases, followed by a variety of O-bases, where NMA refers to N-methylacetamide OCMeNHMe. For each acid–base pair, its interaction energy is displayed, along with the intermolecular distance and the AIM bond critical point density of the XB. The next two columns of Table 1 contain the change caused by the complexation in both the C–X bond length and its vibrational frequency. The final column reports the NMR intermolecular coupling constant between the halogen nucleus and the N or O electron donor atom.

### 2.1. Measures of Halogen Bond Strength

Bromobenzene engages in fairly weak XBs, with interaction energies all less than 3 kcal/mol. The sp-hybridization of the N in NCH provides the weakest bonding, less than 1 kcal/mol, which is enhanced in the sp^2^ hybridization of HN=NH and pyridine. The sp^3^ hybridization of NH_3_ and NMe_3_ leads to the strongest bonding, particularly in the latter, with its three electron-releasing methyl groups. This same electron donating property makes dimethylether a stronger base than water, and the O-bases collectively lead to XB energies between 1.3 and 2.6 kcal/mol. All of the XB energies rise as the Br atom of PhBr is replaced by the more polarizable and electropositive I atom, and a further boost is provided by adding five F substituents to the phenyl ring. The interaction energy range involving PhF_5_I is between 3.6 and 8.7 kcal/mol.

**Figure 2:**
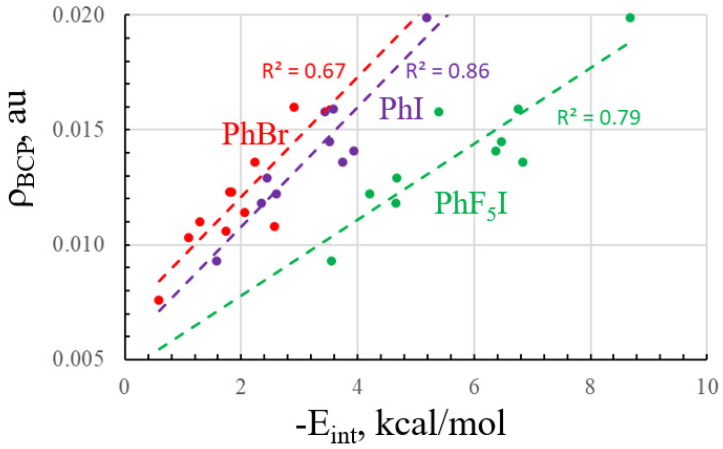
Relationship between interaction energy and X··N/O BCP density.

The intermolecular distances generally reflect the energetic trends. As the XBs strengthen, one sees a shortening of R(X··N/O). The shortest XB of all pairs is PhF_5_I with NMe_3_, with R(I··N) equal to 2.82 Å and an interaction energy of 8.7 kcal/mol. Given their usual tight correspondence with interatomic distance, it is not surprising that the intermolecular AIM bond critical point densities track closely with R. The smallest ρ_BCP_ of 0.0076 au refers to the weak PhBr··NCH complex with R(Br··N) = 3.26 Å, whereas the much shorter 2.82 Å XB in PhF_5_I··NMe_3_ rises up to 0.0199 au. It should be noted parenthetically that several of the complexes bear a second intermolecular bond path. Their densities which are contained in parentheses in Table 1 are fairly small, but not negligible, and they refer to weak H··X H-bonding interactions. Despite these secondary bond paths, the density of the primary X··N/O bond correlates modestly well with the overall interaction energy, as illustrated by the correlation diagram of Figure 2.

The next two columns of Table 1 are suggestive of the perturbing effect of each XB upon the internal C–X covalent bond of the Lewis acid. The weaker XBs tend to induce a small contraction in each such bond and a blue shift in its stretching frequency, i.e., positive Δν. As the bonds strengthen toward the bottom of the table, the pattern shifts toward stretches that are accompanied by red shifts. This reversal of bond characteristics has been explained recently [76] in similar systems in terms of the competition between two effects. While density shifts into the σ*(CX) antibonding orbital tend to weaken and elongate this bond, a contraction which deepens the σ-hole on X would be favored on energetic grounds. The former bond weakening wins this competition when there is a large charge transfer as occurs with the stronger XBs, while the smaller transfers in the weaker XBs allows the C–X contraction to gain the advantage. Other computations [48] have demonstrated there may be a close connection between the C–X bond length in small Lewis acid units such as F_3_CX and the amount by which its stretching frequency changes. Specifically regarding the frequencies, the reader is cautioned that the normal modes in these aromatic systems are not pure C–X stretching motions, but include varying amounts of other nuclear displacement, such as ring distortions. This contamination inhibits the close correspondence between internal bond length and stretching frequency. Indeed, other computations [77] have documented that a blue shift in certain small halogen-bonded complexes such as FCl··CH_3_ can result from a mixing of modes in the two molecules, rather than from electron density shifts.

The 4–6 cm^−1^ red shifts of the C–I stretch are consistent with prior calculations [73] and experimental measurements of the complexes between PhF_5_I and various N bases in solution [66] in the 7–14 cm^−1^ range. Another set of measurements considered the related C–I bond in heptafluoro-2-iodopropane [60] and observed red shifts between 4 and 7 cm^−1^ with aromatic N-bases, closely matching the range observed here for PhF_5_I. A frequency reduction of 19 cm^−1^ was measured when PhF_5_I was paired with a quinuclidine N base [78]. 

A number of other parameters related to the intermolecular bond strengths are collected in Table 2. The first four columns relate the total energy density, H, the potential energy density, V, the Laplacian of the density, and the ellipticity at the intermolecular bond critical point. The next column contains the density at the ring critical point at the approximate center of the ring, which provides a measure of the aromaticity. Another means of assessing this property is E_gap_, the difference in energy between the HOMO and LUMO, both of which are represented by the phenyl π-system. The final column lists E2, the NBO energetic measure of the charge transfer from the lone pair(s) of the electron donor O or N atom into the antibonding σ*(CX) orbital.

The total energy density is rather flat, being mostly positive but only slightly so. V is, of course, negative throughout but does not appear to be closely related to the interaction energy. The density Laplacian is fully positive, suggestive of a closed shell noncovalent interaction. The ellipticity seems to bounce around with no clear pattern. The density of the ring critical point of the phenyl ring shows little variation, around 0.024 for the phenyl system and somewhat lower, at 0.022, for the perfluorinated system. The HOMO–LUMO separation varies slightly more but with no clear association with halogen bond strength. E2, on the other hand, does appear to be closely connected to bond strength. In general, these quantities are poorly correlated with the interaction energy, with correlation coefficients less than 0.4. V and ∇^2^ρ are somewhat better, with R^2^ = 0.87 and 0.78, respectively. The parameter that shows a much stronger linear relationship with the interaction energy is E2, with a correlation coefficient of 0.90.

### 2.2. NMR Data

The last column of Table 1 lists the intermolecular coupling constant between the halogen nucleus and the N or O electron donor of the base. These quantities are positive and negative for N and O, respectively. Although there are indications in the literature [54] that these intermolecular coupling constants tend to correlate with bond strength for various sorts of noncovalent bonds, their magnitudes are only very broadly indicative of E_int_. For example, J(I··N) drops from 278 to 218 Hz when the base coupled with PhI is switched out from NH_3_ to NMe_3_, despite the rise in interaction energy. This moderate to poor correlation has been observed previously for other CI XBs [79] and for the related pnicogen bonds [80].

The remaining NMR data for these monomers and their complexes are organized in Table 3 in much the same way as in Table 1. The first two columns contain the isotropic shielding of the halogen and the C atom to which it is attached within the Lewis acid. As may be seen in the first row of Table 3, this shielding is 2085.9 ppm for the Br of the PhBr monomer, which rises to 3103.7 and 3517.6 ppm for the I atom of PhI and PhF_5_I, respectively. The shielding of the neighboring C also rises in this same progression from 26.2 ppm in PhBr up to 73.3 ppm for PhF_5_I. Also presented in the last three columns are the coupling constants between the pertinent atoms of the acid. The coupling constant for the C–Br bond in PhBr is −67.0 Hz, rising in magnitude for the other two acids up to −270.7 Hz for PhF_5_I. J for the adjacent C–C pair that includes the C to which X is bonded is positive, between 80 and 105 Hz. That for the C–H/C–F bond that is ortho to the halogen atom is quite variable in sign. While J(CH) is positive at 170 Hz, the C–F coupling constant is quite negative at −300 Hz for the same ortho bond in PhF_5_I.

Of particular interest are the perturbations in these NMR spectroscopic quantities upon forming a XB with a base. These changes are listed in the ensuing rows of Table 3 and follow several interesting patterns. Both the X and its neighboring C are deshielded by the interaction to varying degrees. In the case of the X nucleus, Δσ does not strictly adhere to a close relationship with XB strength. The I nucleus of PhI, for example, experiences its smallest change for the very strong bond with NMe_3_, while its largest deshielding occurs for the strong XB with OCHNH_2_ and NMA. In contrast to the behavior of PhI, when in the context of the perfluorinated PhF_5_I acid, the strong bond with NMe_3_ results in a large deshielding of I.

The deshielding of the C atom conforms more closely to the trends of the XB strength, even if imperfectly. Taking PhI as an example, the C nucleus is deshielded by 4.3 ppm in the context of a weak bond with NCH, which builds as the N-base becomes more potent, up to 7.3 ppm with NMe_3_. The relationship with E_int_ is tighter for the perfluorinated acid, for which the C deshielding seems to represent a good indicator of bond strength. A similar sort of correlation was noted previously [74] for a host of substituents other than F when placed on the phenyl ring. It should be noted as well that this enhanced deshielding of the C nucleus conforms to a previous study [81] where a di-iodo perfluorinated benzene was complexed to several anions. The 7 ppm deshielding observed in these strong XBs lies sensibly on the upper range of those computed here for neutral pairs. A similar sort of deshielding accompanies the XB formation of perfluorohalobenzene [82] as well as in iodoalkynes [83]. The chemical shift in C atoms attached to I atoms has been catalogued [68], and the shielding drops as the XB strengthens and the internal C–I bond grows longer, with Δσ varying over a 20 ppm range, easily encompassing the changes of 10 ppm computed above. Another work [72] noted this C is deshielded by 6–7 ppm upon forming a CI··N XB, quite in line with the values computed here. Deshielding of the same magnitude occurs [81,84,85] with other fluorinated iodobenzenes. Deshielding of some 12 ppm has been noted in the acetylenic C≡C-I ^13^C spectrum [70,86], with even larger changes occasioned by the binding to anions [87].

**Figure 3:**
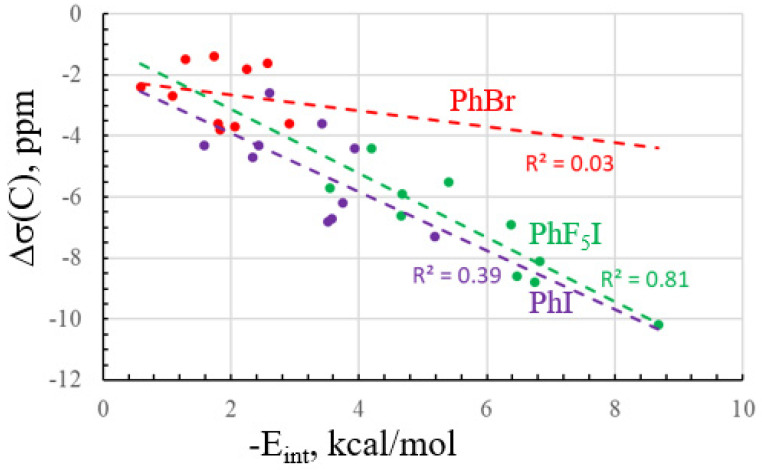
The relationship between interaction energy and change in the isotropic shielding of the C atom bonded to X caused by complexation.

The extent of this relationship is visualized in Figure 3, where a separate line is drawn for each of the three Lewis acids. The red points corresponding to PhBr, with its weak XBs, are rather scattered. The correlation coefficient is quite small, so this NMR parameter would be of little use as a yardstick of XB strength. The stronger XBs containing PhI cover a wider spread, up to 5.2 kcal/mol. The correlation of the purple points in Figure 3 is only marginally better, with R^2^ = 0.39, so the C chemical shift has only limited potential as a gauge of XB strength. The PhF_5_I acid spans an even wider range of energies, and the correlation coefficient is improved to 0.81. It would appear, therefore, that the ability of the C chemical shift to predict the XB energy is best for more potent Lewis acids that cover a wider range of bond strengths.

**Figure 4:**
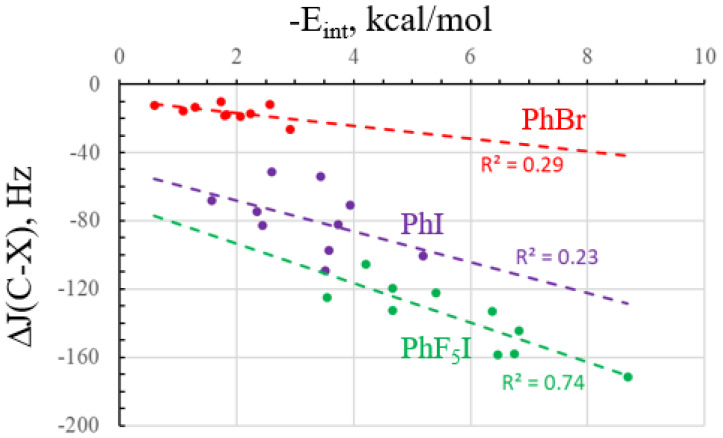
The relationship between the interaction energy and change in the C–X coupling constant caused by complexation.

The coupling constants in the next three columns conform to their own patterns. The largest changes occur within the C–X bonds that interact directly with the base. These quantities are negative and become larger in magnitude as the interaction grows stronger, with certain exceptions. Taking the PhF_5_I acid as an example, the J(CI) coupling constant is −271 Hz. As each N base becomes a more potent nucleophile and yields a stronger XB, this quantity becomes larger in magnitude, swelling by 172 Hz up to −442 Hz for PhF_5_I··NMe_3_. These patterns are evident in Figure 4, which compares ΔJ(CX) with the interaction energy of each dyad. As for Δσ(C), the data for PhBr extends only over a limited range, so the correlation coefficient is poor, at only 0.29. There remains quite a bit of scatter for PhI and no improvement in R^2^. The best correlation is again found in the PhF_5_I systems, where R^2^ climbs to 0.74.

The changes in the other coupling constants are much less dramatic. J(CC) is in the neighborhood of 80–105 Hz and drops a small amount as the XB is formed, less than 2 Hz. There does appear to be a certain correlation, albeit a weak one, between this decrease in J and the XB strength. The coupling constant of the ortho CH/CF bond is about 170 Hz for PhBr and PhI, but a strongly negative −300 Hz for perfluorosubstituted PhF_5_I. In each case, the formation of the XB reduces the magnitude of J. This drop is roughly 1–2 Hz for the first two acids but enlarges to 3–7 Hz for PhF_5_I. 

**Figure 5:**
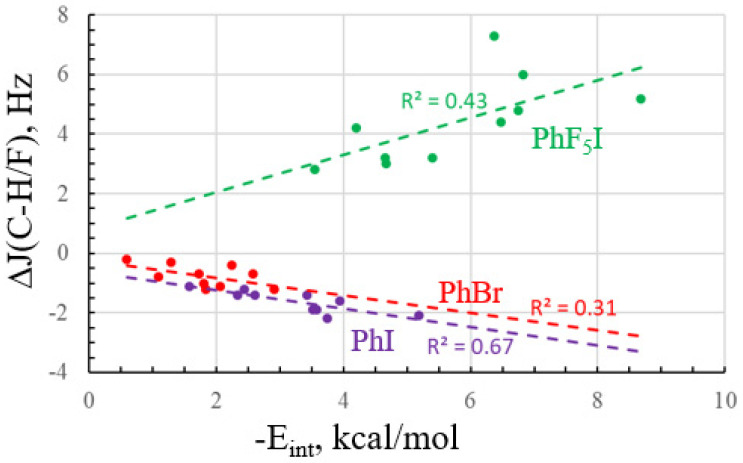
The relationship between the interaction energy and change in the C–H/F coupling constant, ortho to X, caused by complexation.

Like the other coupling constants, J(CH/CF) bears a relation to XB bond strength, but not a highly quantitative one. As is evident in Figure 5, the correlation coefficients range from 0.31 for PhBr up to a maximum of 0.67 for PhI. In the case of this particular parameter, its association with E_int_ is poorer for the stronger PhF_5_I acid. The values of these changes in J(CF) are right in line with changes of 2–4 Hz measured in an earlier study [75].

An earlier examination of the correlations between noncovalent bond strength and spectroscopic features [53] was limited to very small Lewis acids, such as the diatomic FX in the case of halogen bonds. Without the complication of impurity of the F–X stretching frequency arising in a larger molecule such as PhX, linear relationships were found between E_int_ and Δν(FX) with correlation coefficients exceeding 0.9. As noted here, for the larger acids, the relationship with the change in shielding on the X atom was much poorer, at less than 0.7. This work also considered σ-hole bonds other than XBs, but again with small acids such as FHSe and FH_2_As, with similar findings. Other calculations [45] focused on the changes in the IR and NMR spectra resulting from modifications within the acid, keeping the base fixed, and again with small acid molecules. The shift in the X–F bond stretching vibrational frequency diminished as the X atom grew heavier, attributed to this increased mass. In the case of these small FX diatomics, the formation of the XB induced a rise in the X chemical shielding as compared to the reductions observed here for the much larger aromatic systems, with their CX bonds. The replacement of the NH_3_ base by the larger O-base NMA [46] left many of these patterns intact, again limited to the small FX diatomics.

## 3. Methods

Quantum chemical calculations were performed via the density functional approach (DFT) within the context of the M06-2X functional [88], which has been shown to be an accurate means of treating noncovalent bonds of the sort of interest here [89,90,91,92,93,94,95,96,97,98,99]. A polarized triple-ζ def2-TZVP basis set was chosen so as to afford a large and flexible set. Geometries were fully optimized and verified as true minima by the absence of imaginary vibrational frequencies. The Gaussian 16 [100] program was chosen as the specific means to conduct these computations. 

The interaction energy E_int_ of each dyad was calculated as the difference between the energy of the complex and the sum of the energies of the two constituent subunits, each in the geometry they adopt within the dimer. The counterpoise procedure [101] was applied to correct the basis set superposition error of E_int_. Atoms in molecules (AIM) bond paths and their associated critical points were located and their densities evaluated with the aid of the AIMAll program [102]. NMR properties were assessed by the application of the GIAO approach [103,104,105]. In order to allow adjustment of core orbitals to complexation, the def2-TZVP pseudopotential of I was bypassed in the associated NMR calculations, applying instead the all-electron Sapporo-DKH3-TZP-2012-diffuse set [106,107], designed to include certain relativistic effects.

## 4. Conclusions

As has been noted on multiple occasions in the past, the AIM bond critical point density scales closely with halogen bond strength, as well as the intermolecular distance. Analysis of the vibrational normal modes shows a significant mixing of the C–X stretching motion with other nuclear displacements such as phenyl ring distortion. The weakest of the halogen bonds display a C–X bond contraction coupled with a blue shift in the associated frequency, whereas the reverse trends occur for the stronger bonds. The intermolecular X··N/O NMR coupling constant is only partially related to XB strength, with numerous disagreements from one complex to the next. There is better agreement arising from certain internal NMR quantities. The correlation between E_int_ and the shielding change occurring on the C atom bonded to X is poor for the weaker XBs involving PhBr but improves considerably for the stronger XBs involving I, particularly PhF_5_I. The internal C–X coupling constant is likewise best for the most potent Lewis acid, forming the strongest XBs. The coupling constant between the C lying ortho to X and its substituent, whether H or F, also correlates with the XB energy, although not with quantitative accuracy.

## Figures and Tables

**Table 1:** The interaction energies (kcal/mol), intermolecular distance, bond critical point density (au), and intermolecular coupling constant (Hz) within dyads, and the change in C–X bond length (Å) and vibrational stretching frequency (cm^−1^) upon complexation with base.

Base	−E_int_	R(X··N/O)	ρ_BCP_ ^a^	Δr(C-X)	Δν(CX)	J(X··N/O)
PhBr··				1.8957	1109.7	
N≡CH	0.59	3.260	0.0076	−0.0022	5.2	42.1
HN=NH	1.09	3.180	0.0103	−0.0014	5.7	53.0
PyrN	1.80	3.103	0.0123	0.0001	1.3	57.3
NH_3_	1.83	3.124	0.0123	−0.0003	2.8	57.3
NMe_3_	2.91	3.008	0.0160	0.0030	2.4	55.3
OH_2_	1.29	3.089	0.0110	−0.0001	0.6	−58.3
OMe_2_	2.24	2.982	0.0136	0.0011	7.7	−69.7
OCH_2_	1.73	3.101	0.0106 (0.0071)	0.0002	0.8	−43.8
OCHNH_2_	2.57	3.091	0.0108 (0.0086)	0.0016	0.6	−58.5
NMA	2.06	3.007	0.0114 (0.0053)	−0.0019	6.1	−103.6
PhI··				2.0939	1100.5	
N≡CH	1.58	3.284	0.0093	−0.0004	2.4	227.3
HN=NH	2.34	3.221	0.0118	−0.0002	2.2	244.1
PyrN	3.58	3.080	0.0159	0.0026	3.3	267.1
NH_3_	3.52	3.146	0.0145	0.0037	3.6	277.8
NMe_3_	5.18	3.001	0.0199	0.0081	3.9	217.8
OH_2_	2.44	3.100	0.0129	0.0005	3.0	−409.2
OMe_2_	3.43	3.013	0.0158	−0.0002	3.0	−343.8
OCH_2_	2.60	3.143	0.0122	−0.0011	4.4	−256.7
OCHNH_2_	3.94	3.055	0.0141 (0.0079)	0.0019	3.4	−357.2
NMA	3.74	3.022	0.0136 (0.0054)	−0.0005	4.7	−491.2
PhF_5_I··				2.0688	828.3	
NCH	3.55	3.101	0.0093	0.0072	−1.0	334.9
HN=NH	4.66	3.024	0.0118	0.0109	4.7	354.6
Pyr-N	6.75	2.922	0.0159	0.0195	−3.9	354.6
NH_3_	6.47	2.976	0.0145	0.0186	−3.6	377.9
NMe_3_	8.68	2.823	0.0199	0.0303	−6.4	308.9
OH_2_	4.67	2.937	0.0129	0.0081	6.3	−692.9
OMe_2_	5.40	2.890	0.0158	0.0103	5.0	−510.5
OCH_2_	4.20	2.967	0.0122	0.0074	5.7	−406.5
OCHNH_2_	6.37	2.895	0.0141 (0.0079)	0.0126	−1.3	−524.4
NMA	6.83	2.874	0.0136 (0.0054)	0.0107	0.8	−702.5

^a^ (H··X) in parentheses.

**Table 2:** AIM values of the intermolecular bond critical point, density of the ring critical point at the center of an aromatic ring, the LUMO–HOMO energy gap, and NBO E2 for the N/O lone pair → σ*(CX) charge transfer. All values are measured in au except E2, which is measured in kcal/mol.

Base	H	V	∇^2^ρ	Ellipticity	ρ_rcp_	E_gap_	E2
PhBr··							
N≡CH	0.0016	−0.0044	0.0302	0.0017	0.0245	0.306	1.15
HN=NH	0.0015	−0.0061	0.0364	0.0652	0.0245	0.277	1.99
PyrN	0.0015	−0.0076	0.0425	0.0612	0.0268	0.288	2.70
NH_3_	0.0014	−0.0076	0.0414	0.0019	0.0245	0.305	3.48
NMe_3_	0.0012	−0.0103	0.0507	0.0042	0.0245	0.294	3.38
OH_2_	0.0018	−0.0071	0.0427	0.0569	0.0245	0.307	2.45
OMe_2_	0.0020	−0.0094	0.0532	0.0068	0.0245	0.307	2.16
OCH_2_	0.0019	−0.0068	0.0422	0.0007	0.0245	0.296	1.62
OCHNH_2_	0.0018	−0.0067	0.0416	0.0115	0.0245	0.308	2.29
NMA	0.0021	−0.0078	0.0481	0.0208	0.0245	0.304	2.30
PhI··							
N≡CH	0.0016	−0.0054	0.0342	0.0007	0.0246	0.295	1.97
HN=NH	0.0011	−0.0111	0.0536	0.0764	0.0221	0.261	3.00
PyrN	0.0011	−0.0102	0.0499	0.0569	0.0268	0.272	4.74
NH_3_	0.0011	−0.0090	0.0446	0.0001	0.0246	0.296	5.12
NMe_3_	0.0004	−0.0131	0.0557	0.0027	0.0246	0.297	5.43
OH_2_	0.0017	−0.0086	0.0479	0.0746	0.0246	0.296	3.61
OMe_2_	0.0016	−0.0110	0.0568	0.0255	0.0246	0.297	3.33
OCH_2_	0.0017	−0.0077	0.0447	0.0248	0.0246	0.279	2.47
OCHNH_2_	0.0017	−0.0094	0.0514	0.0082	0.0246	0.298	4.12
NMA	0.0020	−0.0095	0.0538	0.0296	0.0245	0.294	3.52
PhF_5_I··							
NCH	0.0017	−0.0084	0.0473	0.0065	0.0220	0.299	3.83
HN=NH	0.0011	−0.0111	0.0537	0.0764	0.0221	0.270	5.87
Pyr-N	0.0006	−0.0146	0.0632	0.0500	0.0222	0.283	8.46
NH_3_	0.0007	−0.0131	0.0578	0.0072	0.0221	0.300	9.01
NMe_3_	−0.0012	−0.0197	0.0693	0.0049	0.0279	0.302	10.58
OH_2_	0.0018	−0.0122	0.0634	0.1057	0.0220	0.296	5.39
OMe_2_	0.0014	−0.0146	0.0692	0.0399	0.0221	0.300	5.69
OCH_2_	0.0017	−0.0117	0.0603	0.0267	0.0220	0.287	4.83
OCHNH_2_	0.0016	−0.0137	0.0675	0.0049	0.0221	0.300	7.26
NMA	0.0019	−0.0136	0.0699	0.0344	0.0221	0.297	6.17

**Table 3:** Values of isotropic shielding (σ, ppm) and coupling constants (J, Hz) in monomers and changes caused by complexation.

Base	Δσ(X)	Δσ(C)	ΔJ(C-X)	ΔJ(C-C)	ΔJ(C-H/F)
PhBr··	2085.9	26.2	−67.0	83.7	170.3
N≡CH	−35.3	−2.4	−12.5	−0.6	−0.2
HN=NH	−43.3	−2.7	−15.6	−0.7	−0.8
PyrN	−44.1	−3.6	−18.2	−1.1	−1.0
NH_3_	−47.0	−3.8	−18.0	−1.3	−1.2
NMe_3_	−52.6	−3.6	−26.7	−1.3	−1.2
OH_2_	−20.8	−1.5	−13.4	−0.7	−0.3
OMe_2_	−44.8	−1.8	−17.2	−0.6	−0.4
OCH_2_	−31.3	−1.4	−10.0	−0.3	−0.7
OCHNH_2_	−34.8	−1.6	−11.7	−0.5	−0.7
NMA	−61.1	−3.7	−19.0	−1.1	−1.1
PhI··	3103.7	39.1	−172.6	80.8	170.7
N≡CH	−77.9	−4.3	−68.2	−1.1	−1.1
HN=NH	−67.6	−4.7	−74.8	−1.3	−1.4
PyrN	−80.8	−6.7	−97.6	−1.6	−1.9
NH_3_	−49.6	−6.8	−109.5	−1.7	−1.9
NMe_3_	−15.4	−7.3	−100.5	−1.9	−2.1
OH_2_	−64.6	−4.3	−83.0	−1.2	−1.2
OMe_2_	−48.5	−3.6	−54.2	−1.1	−1.4
OCH_2_	−77.8	−2.6	−51.6	−0.7	−1.4
OCHNH_2_	−118.1	−4.4	−71.0	−1.1	−1.6
NMA	−120.5	−6.2	−82.4	−1.3	−2.2
PhF_5_I··	3517.6	73.3	−270.7	105.0	−299.5
NCH	−79.8	−5.7	−125.0	−1.2	2.8
HN=NH	−88.8	−6.6	−132.7	−1.3	3.2
Pyr-N	−52.2	−8.8	−157.9	−1.6	4.8
NH_3_	−10.4	−8.6	−158.5	−1.6	4.4
NMe_3_	−82.0	−10.2	−171.6	−1.5	5.2
OH_2_	−75.9	−5.9	−119.7	−1.0	3.0
OMe_2_	−56.5	−5.5	−122.1	−1.2	3.2
OCH_2_ ^a^	−36.4	−4.4	−105.2	−0.8	4.2
OCHNH_2_ ^a^	+0.9	−6.9	−132.9	−1.0	7.3
NMA	−77.5	−8.1	−144.2	−1.2	6.0

^a^ J(CF) for F syn to base.

## Data Availability

Data are contained within the article.
